# Supplementary material for: 3D molecular phenotyping of cleared human brain tissues with light-sheet fluorescence microscopy
Source: Commun Biol. 2022 May 12;5:447. doi: 10.1038/s42003-022-03390-0 (PMC9098858; doi:10.1038/s42003-022-03390-0)
Supplement: Supplementary file 2 — Supplementary information [file 42003_2022_3390_MOESM2_ESM.pdf]

Supplementary Information

3D molecular phenotyping of cleared human brain tissues with light-sheet fluorescence microscopy

Authors
Luca Pesce[1,2], Marina Scardigli[1,2], Vladislav Gavryusev[1,2], Annunziatina Laurino[1,7], Giacomo Mazzamuto[1,3], Niamh Brady[1], Giuseppe Sancataldo[1], Ludovico Silvestri[1-3], Christophe Destrieux[4], Patrick R. Hof[5], Irene Costantini*[1,3,6], Francesco S. Pavone[1,2,3]

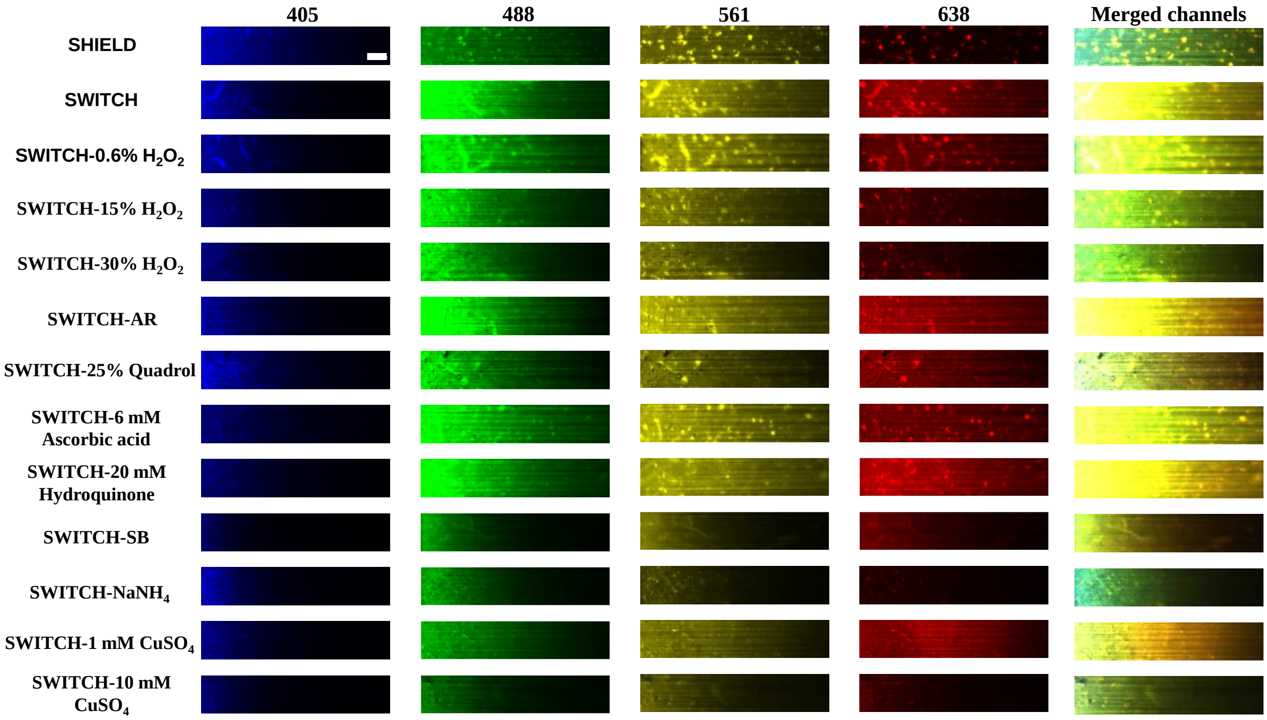

Supplementary Figure 1. Characterization of SWITCH processed human brain slices treated with different autofluorescence elimination reagents. Columns 1-4 correspond to the 4 different excitation lights; column 5 correspond to the merge channels for each treatment. The look-up tables (LUTs) are fixed for each treatment. Scale bar = 100 μm.

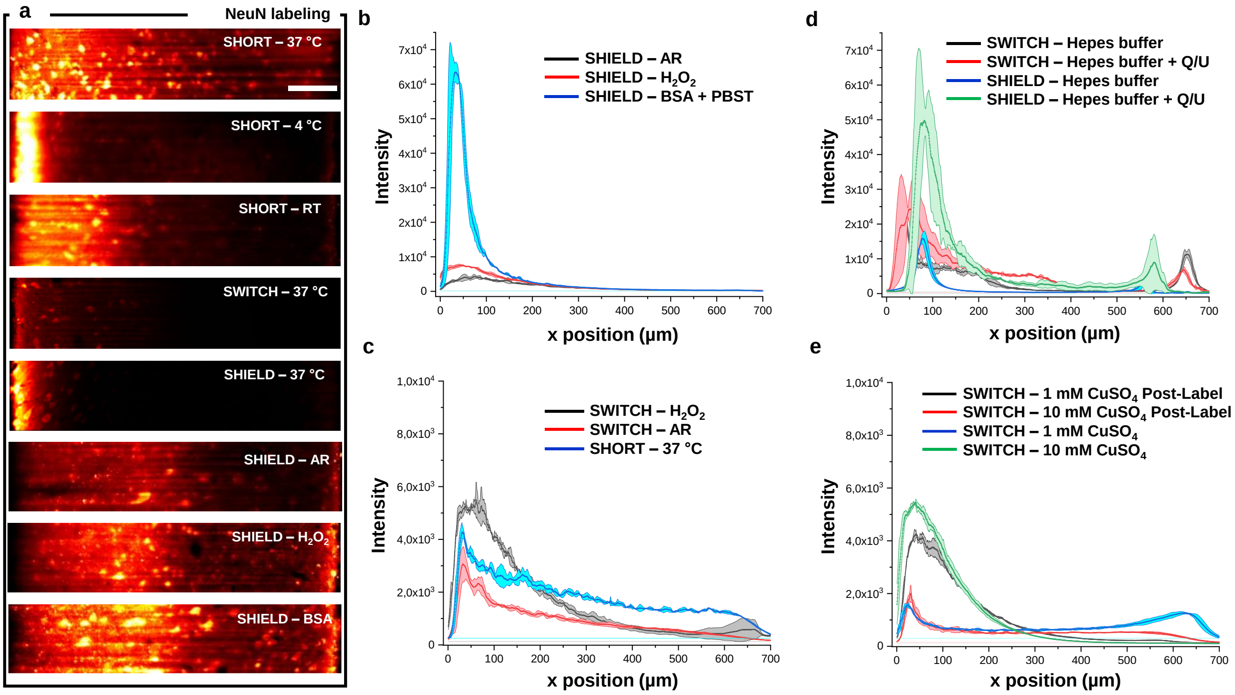

Supplementary Figure 2. Effect of tissue processing (SWITCH, SHIELD, and SHORT), autofluorescence treatments ($CuSO_4$), and buffer on NeuN fluorescence labeling. a High resolution imaging of NeuN labeling of different experiments shown in Fig. 1. Scale bar = 100 μm. **b** Profile plots of NeuN labeling of SHIELD-processed slices incubated with BSA + PBST, treated with AR or H_2O_2. (**c**) Comparison of SWITCH-processed slices treated with H_2O_2, AR or by combining both (SHORT). **d** Profile plots of NeuN antibody incubated with HEPES buffer, or HEPES buffer supplemented with 2.5% Quadrol and 0.5 M urea in SWITCH/SHIELD-processed slices. **e** Profile plots of SWITCH-processed slices treated with 1 mM $CuSO_4$ after NeuN labeling (black and red profiles) or before NeuN labeling (blue and green profiles). Antibodies dilution for all experiments: NeuN 1:100; Alexa Fluor 647 1:500. Incubation temperature of 37 °C (N = 3). Excitation light, 638 nm at a 5 mW laser power.

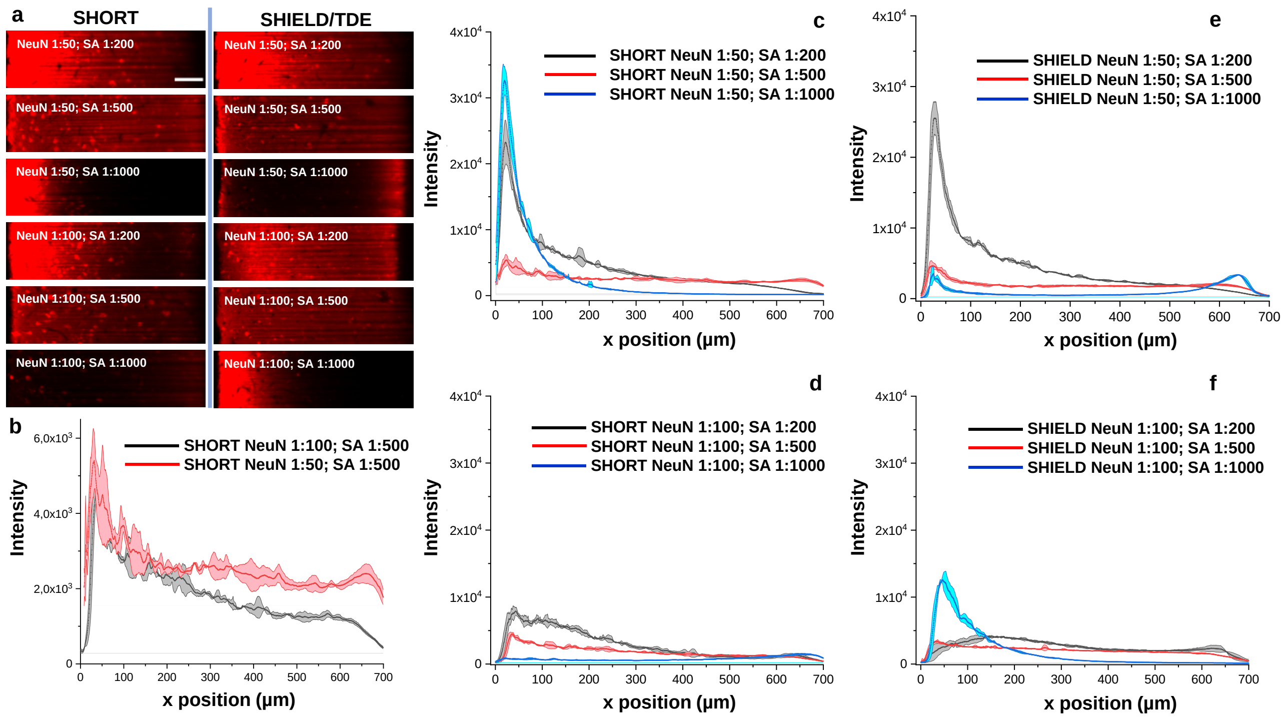

Supplementary Figure 3. Antibody optimization of SHORT and SHIELD-processed slices. a High resolution images acquired by LSFM of SHORT and SHIELD-processed slices, labeled for NeuN with Alexa Fluor 647 at different dilution (1:50 and 1:100 for NeuN; from 1:200 to 1:1000 for Alexa Fluor 647). Scale bar = 100 µm. **b** Profile plot along depth of the two best conditions of NeuN and Alexa Fluor 647 antibodies in SHORT-processed tissues. **c-d** Profile plots of different antibody dilutions of SHORT-processed slices. **e-f** Profile plot of different antibody dilutions of SHIELD-processed slices. Incubation temperature of 37 °C (n = 3). Excitation light, 638 nm; laser power, 5 mW.

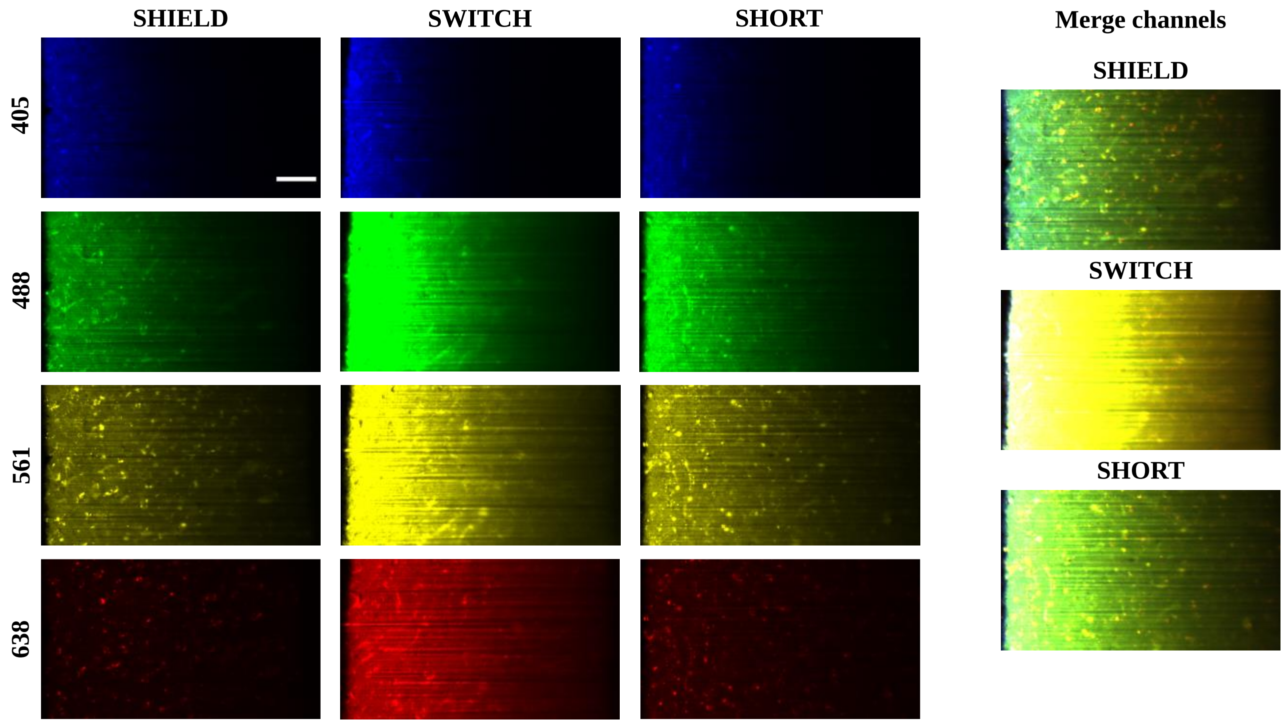

Supplementary Figure 4. Comparison of autofluorescence signal between SHIELD, SWITCH and SHORT. SWITCH-, SHORT-, and SHIELD-processed slices excited at 405, 488, 561 and 638 nm. SHIELD and SHORT show similar autofluorescence signal at 405, 561, and 638 nm, while SHORT shows higher intensity at 488 nm (see also Fig. 1A). Laser power, 5 mW. The LUTs are fixed for each treatment. Scale bar = 100 µm.

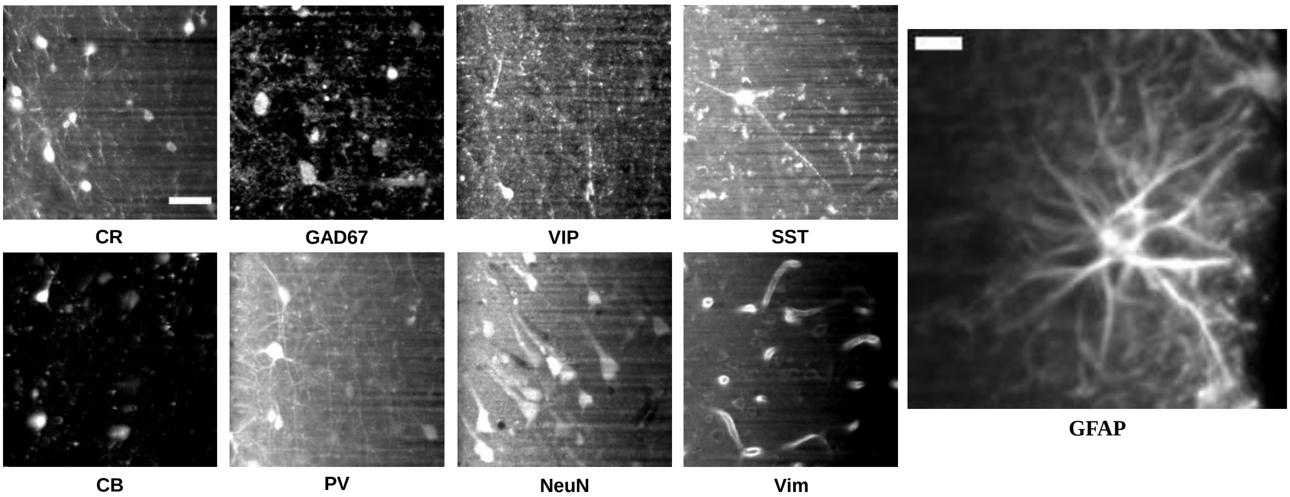

Supplementary Figure 5. High-resolution images acquired by LSFM (resolution of 0.55 x 0.55 x 3.3 µm). Representative images showing morphological details of calretinin (CR)-, glutamic acid decarboxylase 67 (GAD67)-, vasoactive intestinal peptide (VIP)-, somatostatin (SST)-, calbindin (CB)-, parvalbumin (PV), neuronal nuclear protein (NeuN)-, glial fibrillary acidic protein (GFAP)-immunoreactive cortical neurons, and vimentin (VIM) immunolabeling of blood vessels. Scale bar = 100 µm for all images, except for GFAP (scale bar 10 = µm).

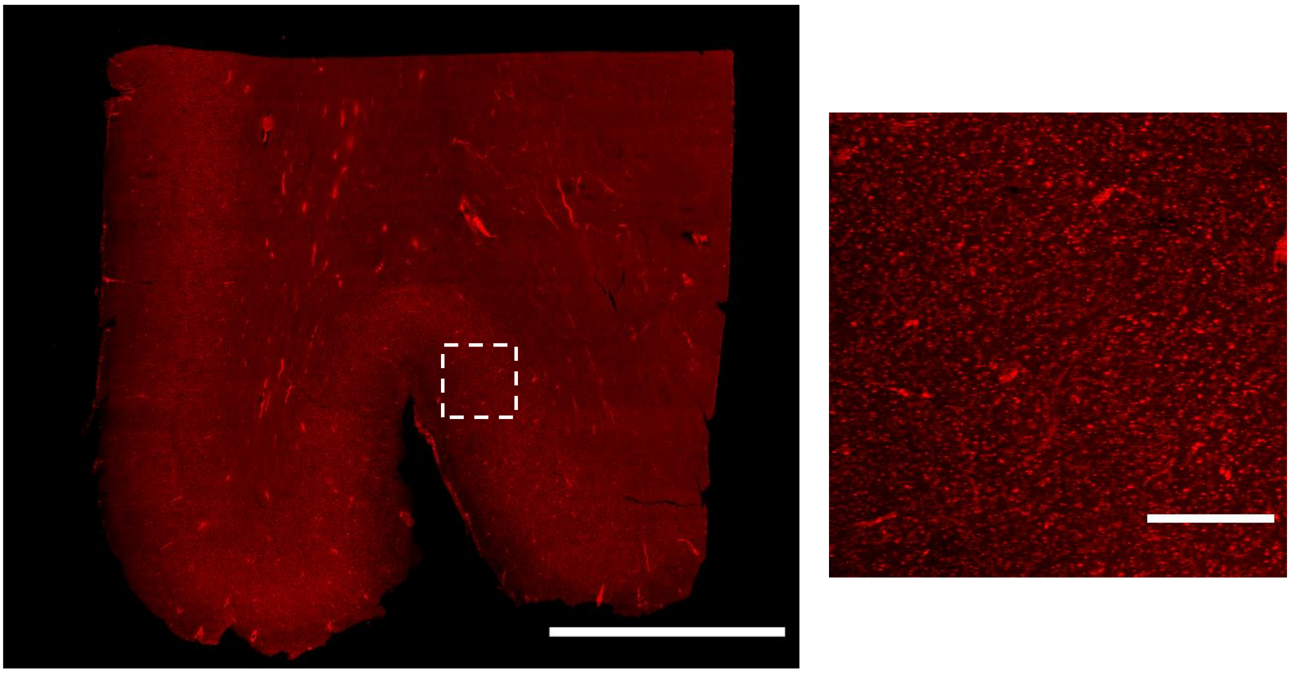

Supplementary Figure 6. Propidium iodide (PI) allows an efficient nuclear labeling in SHORT-processed samples. Human motor cortex labeled with PI and acquired using LSFM. MIP of 20 slices. Excitation light 561 nm. Scale bar = 4 mm. Magnified image of the nuclear staining, scale bar = 200 μm.

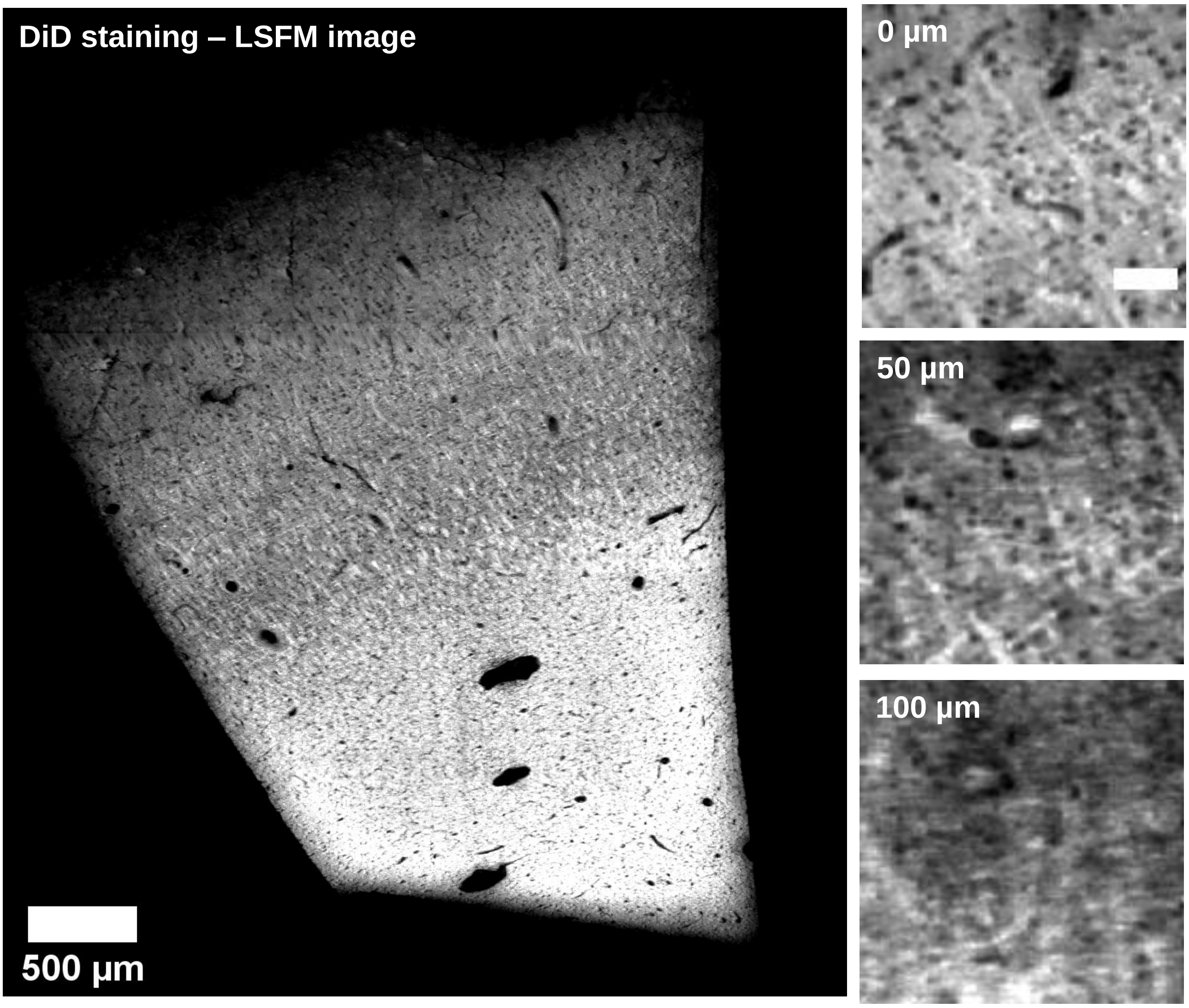

Supplementary Figure 7. DiD staining on a SHORT-processed human brain slice. Downscaled images of the precentral cortex (100 µm-thick) and the intensity signal of the fiber stained with DiD. SHORT is compatible with lipophilic dye and the signal along the thickness is sufficient to detect the labeled fibers. Excitation light, 638 nm; laser power, 1 mW. Scale bar = 10 µm.

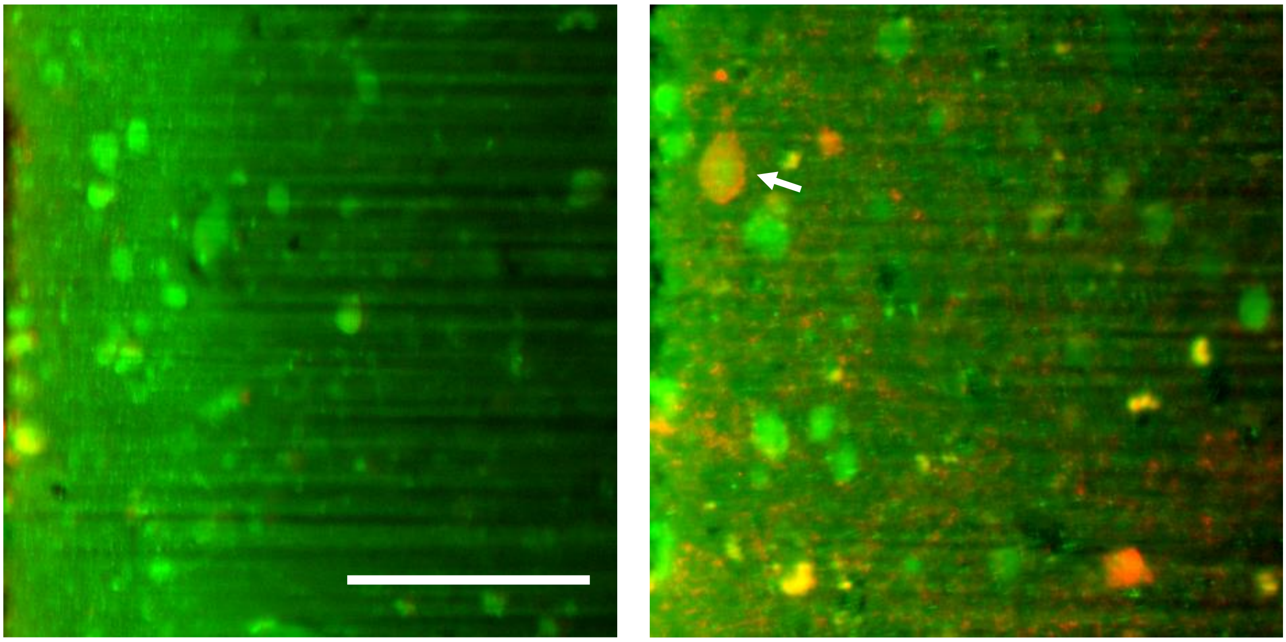

Supplementary Figure 8. Comparison between the classic SWITCH protocol and SHORT for NeuN (green) - GAD67 (red) costaining. The white arrow shows the high-resolution imaging of a typical costained neuron positive for NeuN and GAD67 in SHORT-processed slices. Scale bar = 100 µm. Excitation light: 488 nm and 638 nm.

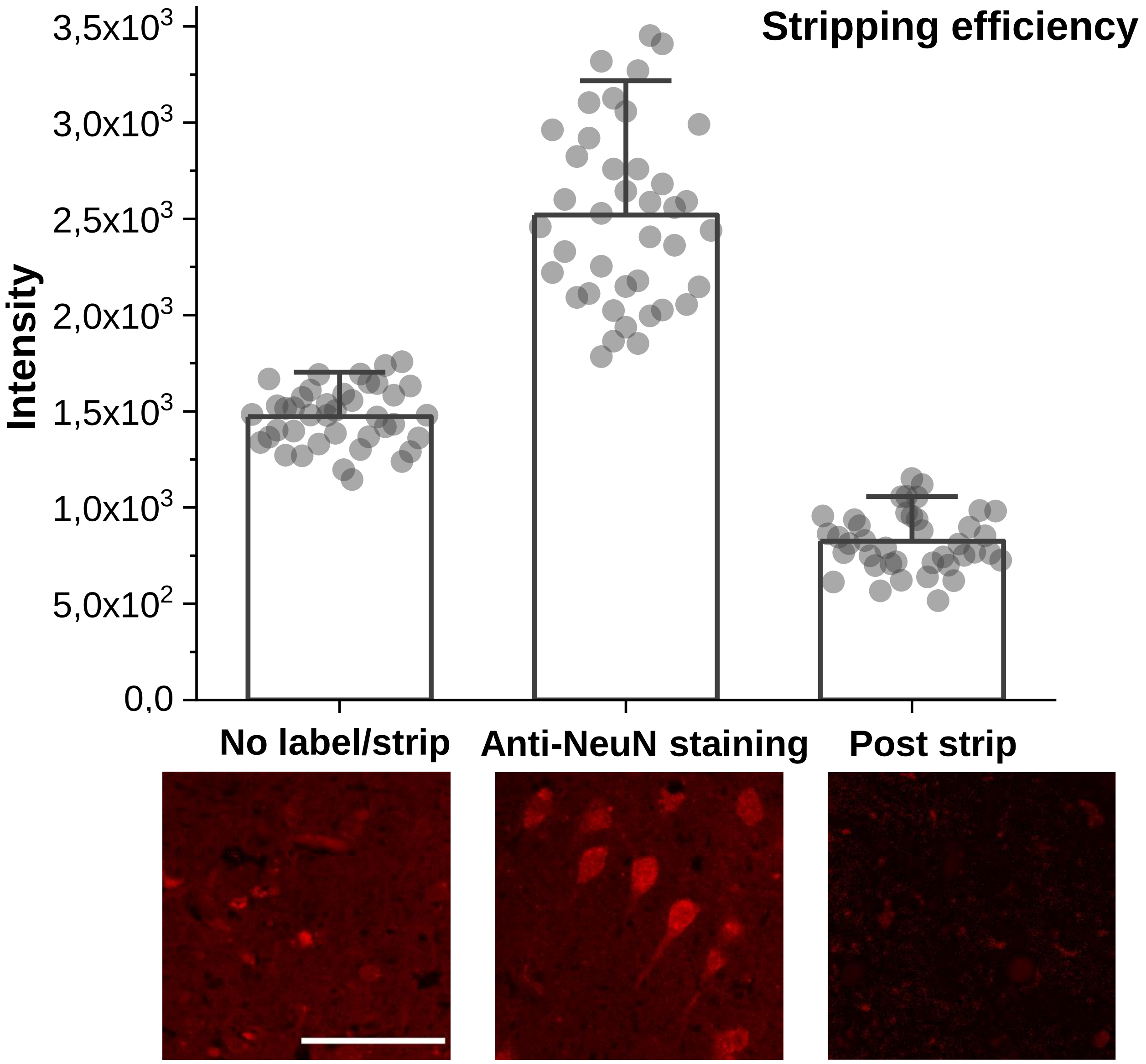

Supplementary Figure 9. Stripping efficiency in SHORT-processed human slices. Tissue sections were processed with SHORT and were acquired using a Nikon C2 laser-scanning confocal microscope. Then, the samples were immunostained for NeuN with AlexaFluor 568 and were re-acquired by the same microscope. The samples were then stripped using the elution buffer (see Methods) for 4 °h at 80 °C and were acquired again. Quantification of fluorescence intensity seen in n = 3 samples. Error bars: standard deviation. Scale bar = 50 μm.

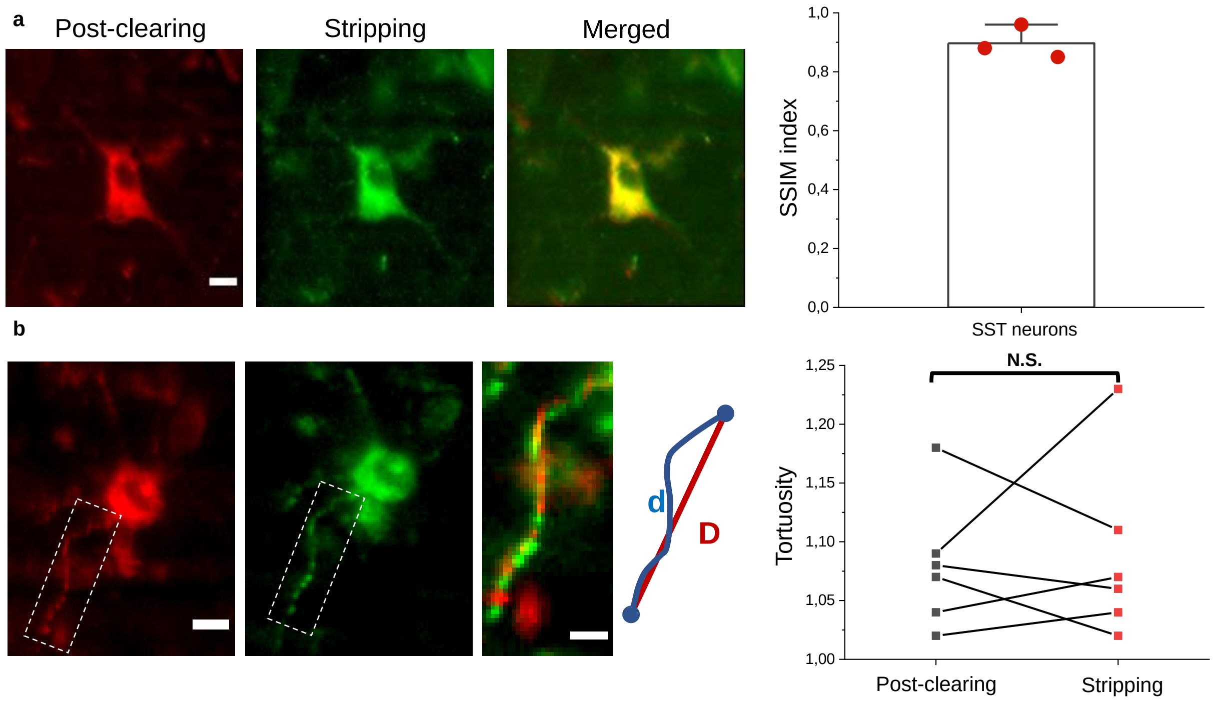

Supplementary Figure 10. Distortion analysis. SHORT-processed human cortical slice immunostained for SST after the clearing process (post-clearing, red images). Then, the antibodies were stripped and restained for SST (stripping, green image). **a** Comparison of the structural similarity between two high-resolution images of SST-immunoreactive neurons after clearing and stripping process using the structural similarity index measure (SSIM index; range value between -1 and 1; two identical images have an SSIM index close to 1). The data were shown as the mean ± SD (N = 3). **b** Comparison of tortuosity of SST branches between high-resolution images post-clearing (red) and after stripping (red). The minimum distance (D) and the real branch length (d) between the two ends of each individual branch were measured to evaluate the morphological changes using the torsional index (d/D). The branch alteration after the stripping process is not statically significant (N.S.; N = 6). LSFM images; scale bar = 10 μm; scale bar of the merged image of SST branch = 5 μm.

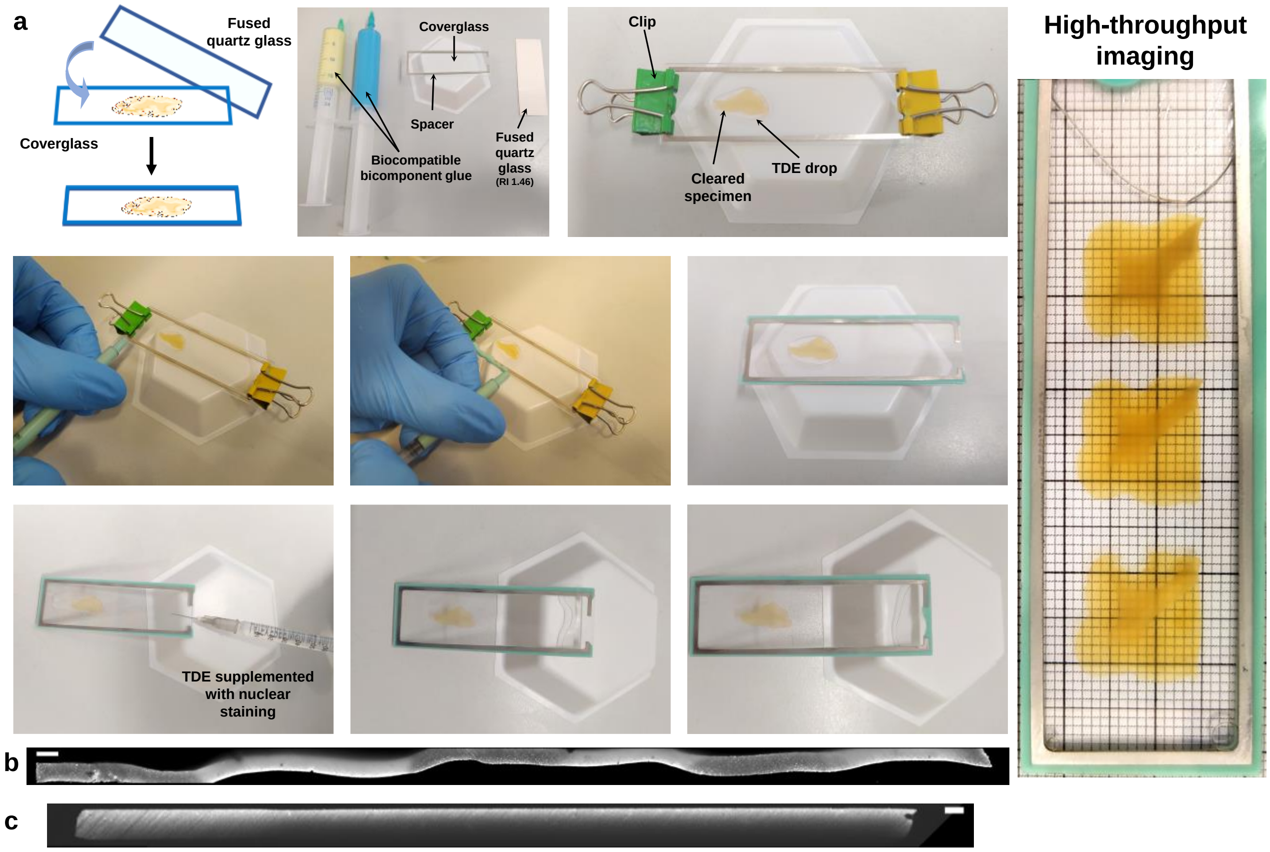

Supplementary Figure 11. Sample holder assembly. a The sandwich holder consists of a cover glass, a spacer (thickness of 500 μm) and fused silica glass. The cleared specimen is fixed between the cover glass and the fused silica glass using a bicomponent glue. Such strategy allows soaking the sample in the TDE/PBS solution supplemented with a nuclear dye, and performing high-throughput imaging of several labelled human slices. Resliced images of mesoscopic reconstruction acquired by LSFM using the classic fused silica glass (**b**) upon the slice or the sandwich apparatus (**c**). Scale bar = 1 mm.

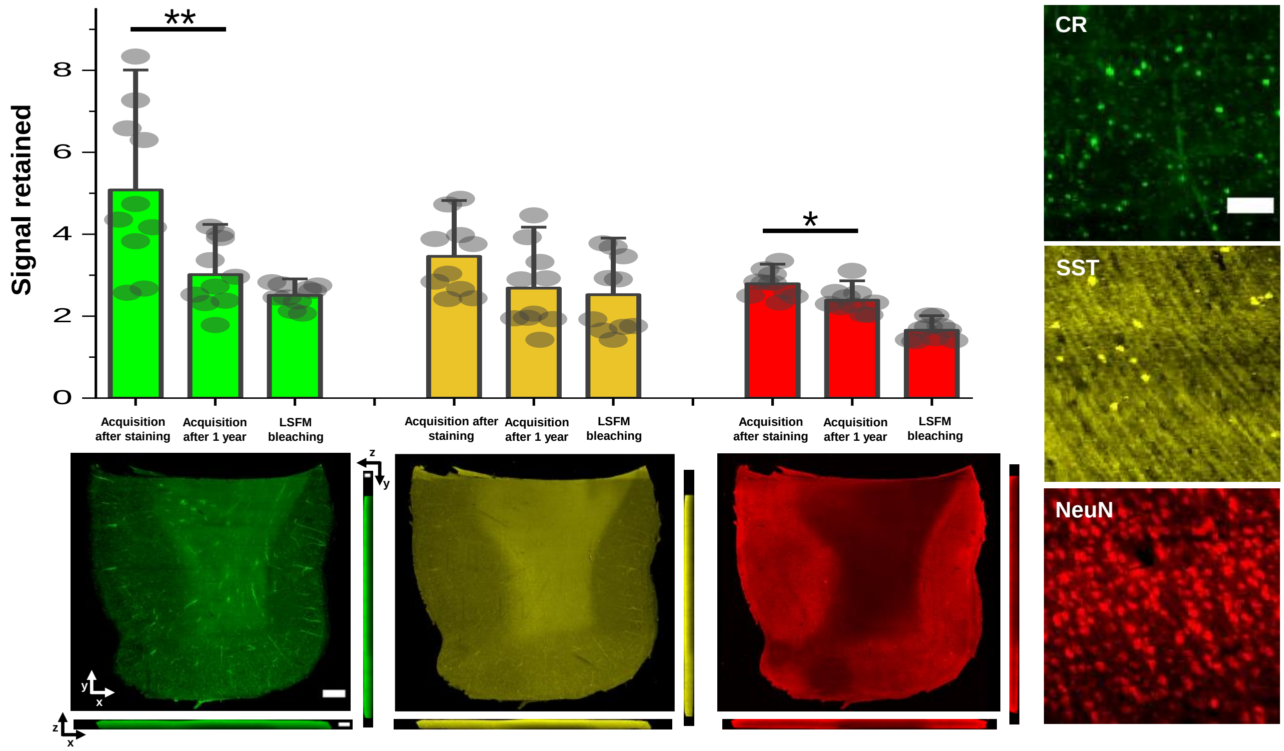

Supplementary Figure 12. Signal retention (signal/background) after 1 year in the sandwich holder. Quantification of the signal retention of 10 neurons acquired after immunostaining (Fig. 4l-n), and then re-acquired after 1 year, and 1 year+1 week kept in the sandwich holder. The data show a reduction of the fluorescence signal of 51% for Alexa Fluor 488 ($P < 0.01$), 24% for Alexa Fluor 568 (no significant), and 14% for Alexa Fluor 647 ($P < 0.05$) (Mann-Whitney test). Scale bar xy = 1000 µm; scale bar xz = 500 µm; scale bar yz = 250 µm. The magnified image below the downscaled reconstruction highlights that after a year of storage the markers are well detectable. Scale bar = 100 µm.

Shrinking effect	PBS (cm^2)	SHORT (cm^2)
Sample 1	7.188	7.048
Sample 2	7.181	6.92
Sample 3	7.396	7.027

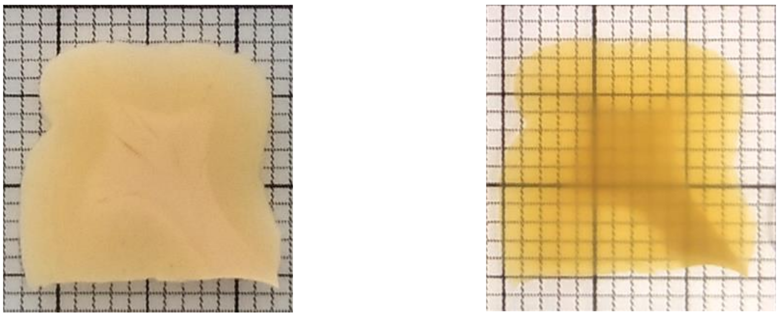

Supplementary Figure 13. Shrinking characterization of SHORT processed slices after incubation in the 68% TDE/PBS solution (N = 3 samples). The slices to be enclosed into the holder, were equilibrated in the TDE solution, tuned for the refractive index matching of the delipidated sample (RI ≈ 1.46 with TDE/PBS solution at 68%), minimizing optical aberration and making the sample almost completely transparent. After equilibration of the processed sample into the PBS/TDE solution, we observed an isotropic tissue shrinkage of a factor of 3.5 ± 1.5 due to the RI matching obtained with the organic solvent TDE. Generally, the TDE solution is supplemented with nuclear staining.

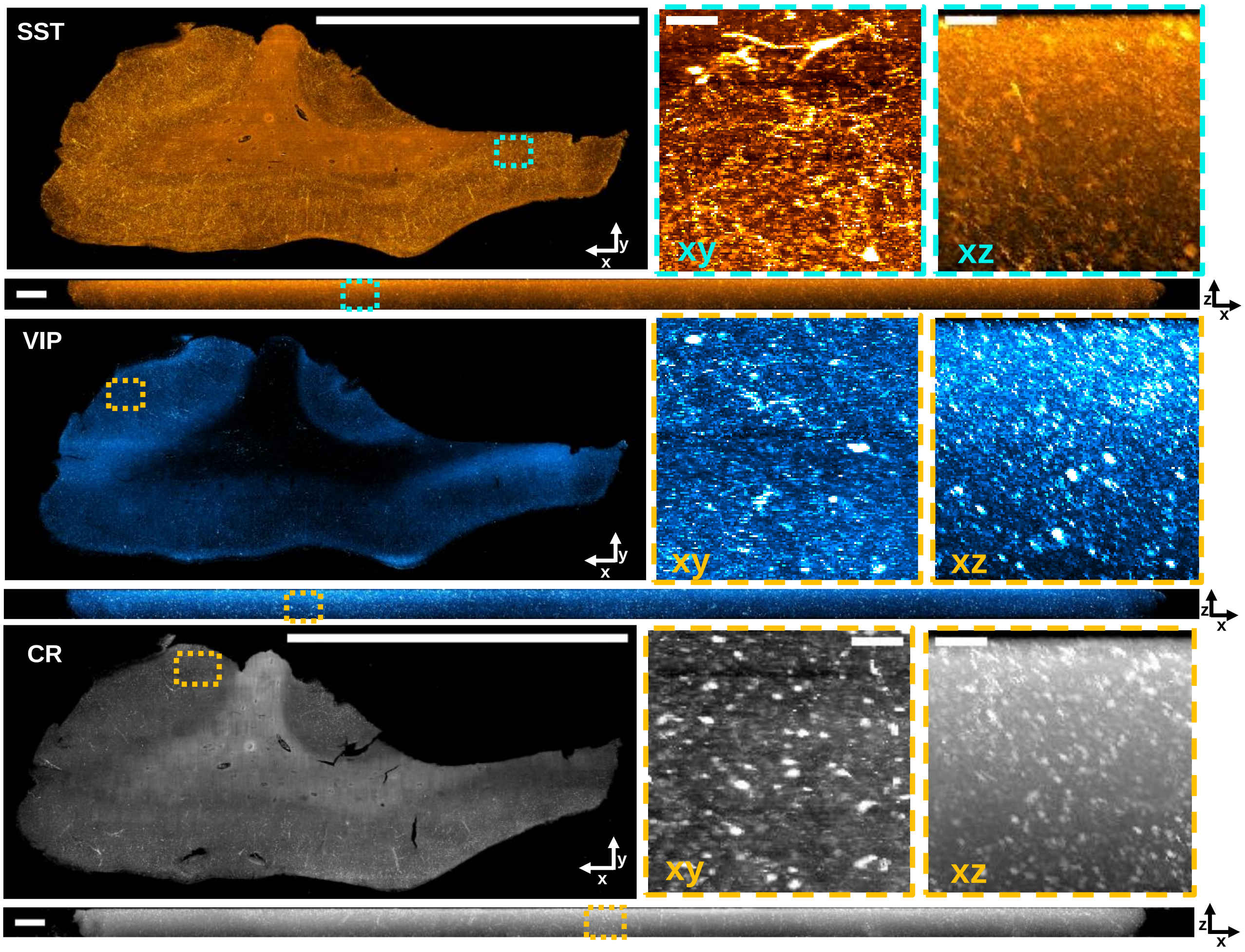

Supplementary Figure 14. Multiplexed staining of SHORT-processed slice and the homogenous staining along the thickness. xy and zx MIP images of the superior frontal cortex stained for SST (Alexa Fluor 568; orange), VIP (Alexa Fluor 647; blue), and CR (Alexa Fluor 488; grey). MIPs of 150 slices (whole thickness). Scale bar xy images = 1 cm; scale bar xz = 500 μm; scale bar of the magnified insets = 100 μm.

Molecule	Company	Cat. n.	Host	P/M	Dilution
NeuN	Merck	ABN91	Chicken	P	1:50
GAD67	Abcam	ab26116	Mouse	M	1:200
PV	Abcam	ab11427	Rabbit	P	1:200
PV	Abcam	ab32895	Goat	P	1:200
CB	Abcam	ab207528	Rabbit	M	1:200
VIP	Abcam	ab214244	Rabbit	M	1:200
SST	Abcam	ab30788	Rat	M	1:200
NPY	Abcam	ab6173	Sheep	P	1:200
NPY	Abcam	ab11247	Mouse	M	1:200
SMI-32	Merck	NE1023	Mouse	M	1:200
SMI-31	Eurogentec OptimAb	SMI-31P-050	Mouse	M	1:300
Neurofilament	Abcam	ab4680	Chicken	P	1:200
GluS	Merck	MAB302	Mouse	M	1:200
MAP2	Abcam	ab5392	Chicken	P	1:200
GFAP	Abcam	ab194324	Rabbit	M	1:200
Iba1	Abcam	ab195031	Rabbit	M	1:200
Coll IV	Abcam	ab6586	Rabbit	P	1:200
Vim	Abcam	ab8069	Mouse	M	1:200
CR	Proteintech	66496-1-Ig	Mouse	M	1:200
CR	Proteintech	12278-1-AP	Rabbit	P	1:200
Anti-Rat IgG, AF 568	Abcam	ab175475	Donkey	P	1:200
Anti-Rabbit IgG, AF 568	Abcam	ab175470	Donkey	P	1:200
Anti-Chicken IgY, AF 568	Abcam	ab175711	Goat	P	1:200
Anti-Mouse IgG, AF 568	Abcam	ab175700	Donkey	P	1:200
Anti-Sheep IgG, AF 568	Abcam	ab175712	Donkey	P	1:200
Anti-Rabbit IgG, AF 488	Abcam	ab150077	Goat	P	1:200
Anti-Chicken IgY, AF 488	Abcam	ab150169	Goat	P	1:200
Anti-Rabbit IgG, AF 488	Jackson	611-545-215	AffiniPure Alpaca	P	1:200
Anti-Goat IgG, AF 488	Jackson	805-545-180	AffiniPure Bovine	P	1:200
Anti Rabbit IgG, AF 647	Jackson	611-605-215	AffiniPure Alpaca	P	1:200
Anti Mouse IgG, AF 647	Abcam	ab150107	Donkey	P	1:200
Anti Chicken IgY, AF 647	Abcam	ab150171	Goat	P	1:200
DiD	Thermo Fisher Scientific	D7757			0.5 mg/ml
DAPI (Dilactate)	Thermo Fisher Scientific	D3571			1:50
SYTOX GREEN	Thermo Fisher Scientific	S7020			1:100
Propidium Iodide	Thermo Fisher Scientific	P3566			1:50

Supplementary Table 1. Antibodies compatible with SHORT and the used dilutions.

	Area	Age	Fixative	Fixation
**Donor 1**	- Precentral gyrus<br>- Hippocampus	99 years old	10% Formalin	6 months
**Donor 2**	- Prefrontal cortex	62 years old	10% Formalin	4 years
**Donor 3**	- Broca's area<br>- Motor cortex	79 years old	10% Formalin	10 years

Supplementary Table 2. Area, age, fixative molecule, and fixation of the human brain material used in this work.
