# Supplementary material for: 3D molecular phenotyping of cleared human brain tissues with light-sheet fluorescence microscopy
Source: Commun Biol. 2022 May 12;5:447. doi: 10.1038/s42003-022-03390-0 (PMC9098858; doi:10.1038/s42003-022-03390-0)
Supplement: Supplementary file 3 — Description of Additional Supplementary Files [file 42003_2022_3390_MOESM3_ESM.pdf]

Description of Additional Supplementary Files

File name: Supplementary Movie 1
Description: High resolution stacks of SHORT-processed slices acquired using LSFM (resolution of 0.55 x 0.55 x 3.3 µm) labelled for CR with Alexa Fluor 488

File name: Supplementary Movie 2
Description: High resolution stacks of SHORT-processed slices acquired using LSFM (resolution of 0.55 x 0.55 x 3.3 µm) labelled for SST with Alexa Fluor 568

File name: Supplementary Movie 3
Description: High resolution stacks of SHORT-processed slices acquired using LSFM (resolution of 0.55 x 0.55 x 3.3 µm) labelled for NeuN with Alexa Fluor 647.

File name: Supplementary Movie 4
Description: Downscaled reconstruction of the Broca's area labelled for NeuN with Alexa Fluor 647. Resolution of 3.3 x 3.3 x 3.3 µm.
