# Supplementary figures and images for: 3D molecular phenotyping of cleared human brain tissues with light-sheet fluorescence microscopy
Source: Commun Biol. 2022 May 12;5:447. doi: 10.1038/s42003-022-03390-0 (PMC9098858; doi:10.1038/s42003-022-03390-0)

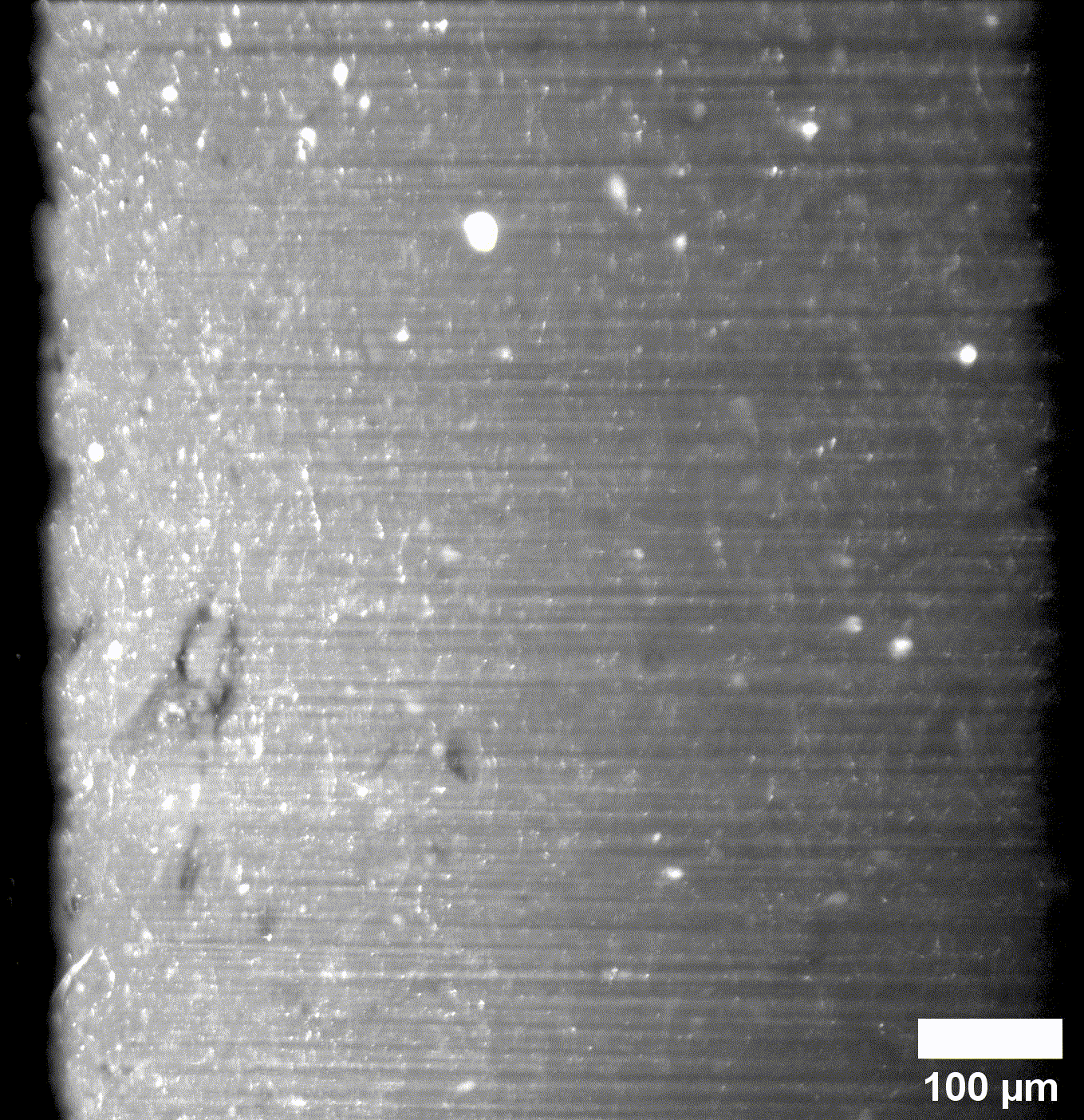

Supplement: Supplementary file 4 — Supplementary movie 1 [file 42003_2022_3390_MOESM4_ESM.gif]

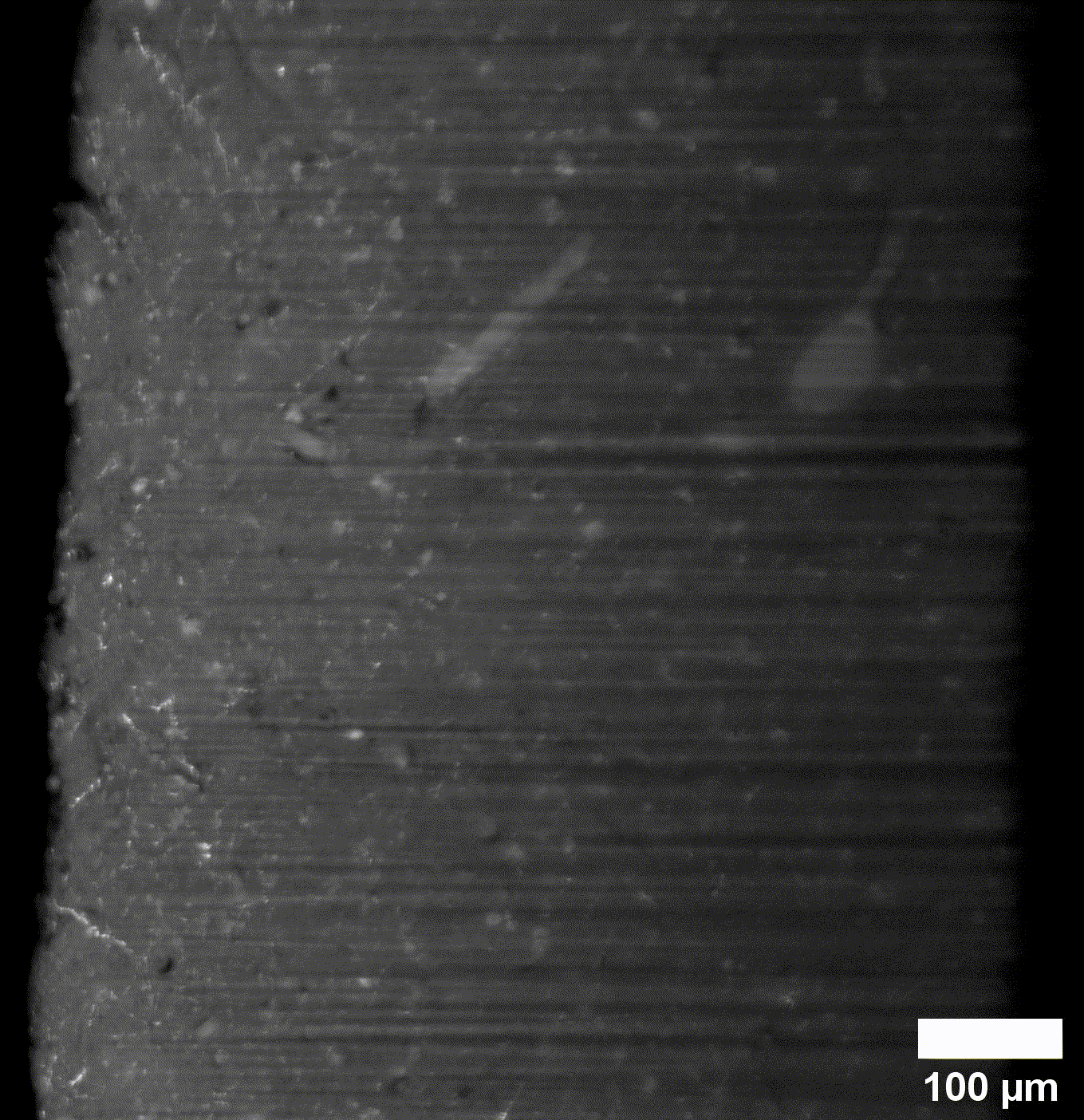

Supplement: Supplementary file 5 — Supplementary movie 2 [file 42003_2022_3390_MOESM5_ESM.gif]

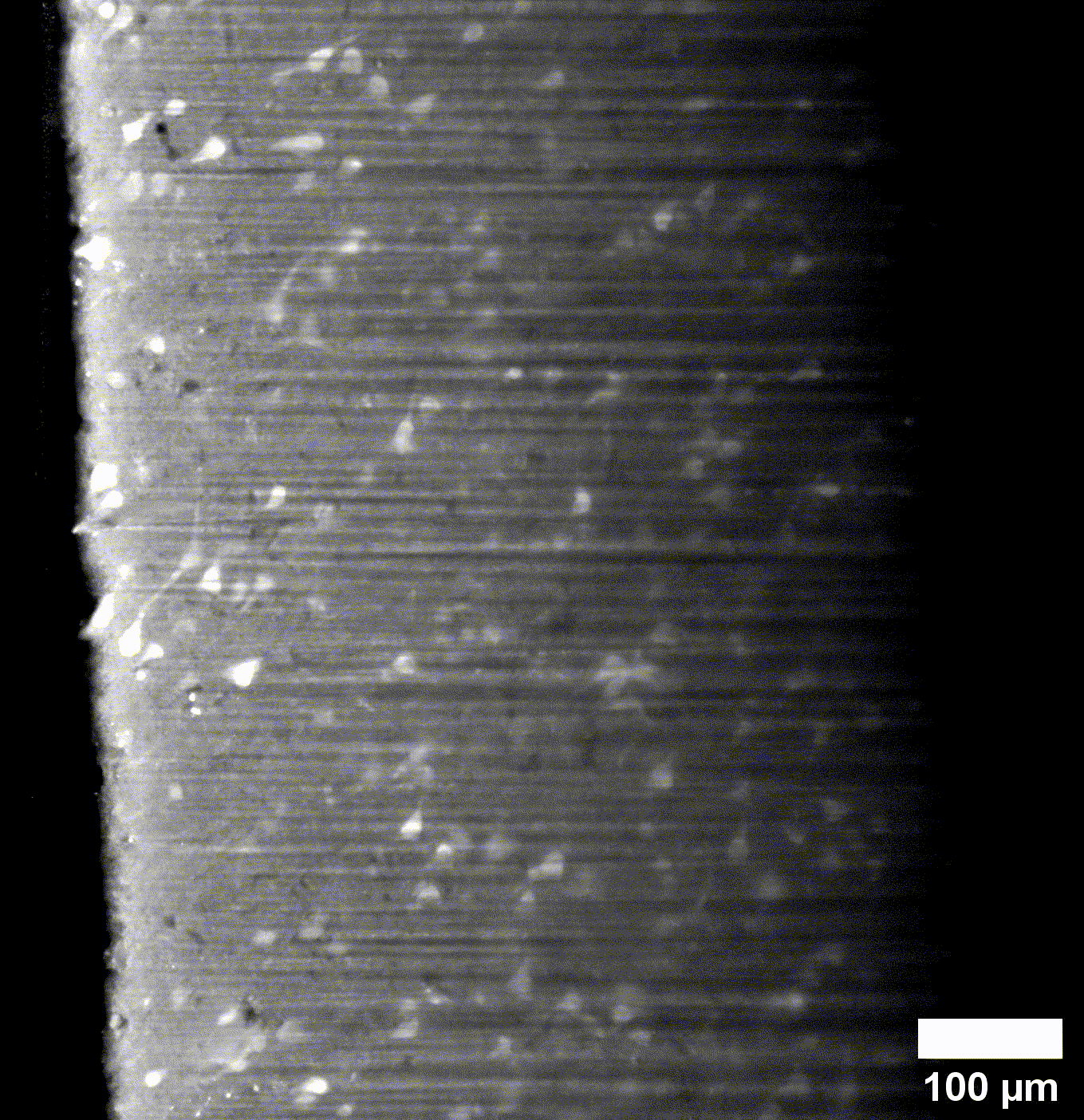

Supplement: Supplementary file 6 — Supplementary movie 3 [file 42003_2022_3390_MOESM6_ESM.gif]
