# Supplementary material for: 3D molecular phenotyping of cleared human brain tissues with light-sheet fluorescence microscopy
Source: Commun Biol. 2022 May 12;5:447. doi: 10.1038/s42003-022-03390-0 (PMC9098858; doi:10.1038/s42003-022-03390-0)
Supplement: Supplementary file 8 — Reporting Summary [file 42003_2022_3390_MOESM8_ESM.pdf]

nature portfolio

Corresponding author(s): Irene Costantini

Last updated by author(s): Mar 9, 2022

Reporting Summary

Nature Portfolio wishes to improve the reproducibility of the work that we publish. This form provides structure for consistency and transparency in reporting. For further information on Nature Portfolio policies, see our Editorial Policies and the Editorial Policy Checklist.

Statistics

For all statistical analyses, confirm that the following items are present in the figure legend, table legend, main text, or Methods section.

n/a	Confirmed	
☐	☒	The exact sample size (*n*) for each experimental group/condition, given as a discrete number and unit of measurement
☐	☒	A statement on whether measurements were taken from distinct samples or whether the same sample was measured repeatedly
☐	☒	The statistical test(s) used AND whether they are one- or two-sided<br>*Only common tests should be described solely by name; describe more complex techniques in the Methods section.*
☐	☒	A description of all covariates tested
☐	☒	A description of any assumptions or corrections, such as tests of normality and adjustment for multiple comparisons
☐	☒	A full description of the statistical parameters including central tendency (e.g. means) or other basic estimates (e.g. regression coefficient) AND variation (e.g. standard deviation) or associated estimates of uncertainty (e.g. confidence intervals)
☐	☒	For null hypothesis testing, the test statistic (e.g. *F*, *t*, *r*) with confidence intervals, effect sizes, degrees of freedom and *P* value noted<br>*Give P values as exact values whenever suitable.*
☒	☐	For Bayesian analysis, information on the choice of priors and Markov chain Monte Carlo settings
☐	☒	For hierarchical and complex designs, identification of the appropriate level for tests and full reporting of outcomes
☒	☐	Estimates of effect sizes (e.g. Cohen's *d*, Pearson's *r*), indicating how they were calculated

Our web collection on statistics for biologists contains articles on many of the points above.

Software and code

Policy information about availability of computer code

Data collection	All the data collected are made available on the DANDI platform.
Data analysis	For data analysis, we used Python, Origin 2019, and Fiji

For manuscripts utilizing custom algorithms or software that are central to the research but not yet described in published literature, software must be made available to editors and reviewers. We strongly encourage code deposition in a community repository (e.g. GitHub). See the Nature Portfolio guidelines for submitting code & software for further information.

Data

Policy information about availability of data

All manuscripts must include a data availability statement. This statement should provide the following information, where applicable:

- Accession codes, unique identifiers, or web links for publicly available datasets
- A description of any restrictions on data availability
- For clinical datasets or third party data, please ensure that the statement adheres to our policy

All the datasets acquired for this study are made available on the DANDI platform at the link: https://gui.dandiarchive.org/#/dandiset/000026. All data needed to evaluate the conclusions in the paper are present in the paper and/or the Supplementary Materials. Raw data other than the representative images are available from the corresponding author upon reasonable request

Field-specific reporting

Please select the one below that is the best fit for your research. If you are not sure, read the appropriate sections before making your selection.

☒ Life sciences ☐ Behavioural & social sciences ☐ Ecological, evolutionary & environmental sciences

For a reference copy of the document with all sections, see nature.com/documents/nr-reporting-summary-flat.pdf

Life sciences study design

All studies must disclose on these points even when the disclosure is negative.

Sample size	Approximately 1.5 x 1.5 x 0.5 cm^3
Data exclusions	*Describe any data exclusions. If no data were excluded from the analyses, state so OR if data were excluded, describe the exclusions and the rationale behind them, indicating whether exclusion criteria were pre-established.*
Replication	Several independent experiments were performed to guarantee the reproducibility of the findings. For autofluorescence analysis n = 20; for the the NeuN depth analysis n = 3; quantification of antibodies removal n = 4; Protein retention during the stripping process n=30
Randomization	The brain tissue specimens were taken from 3 different human body donors. To quantify the sample characteristics, regions of interest were selected randomly in the grey and white matter.
Blinding	*Describe whether the investigators were blinded to group allocation during data collection and/or analysis. If blinding was not possible, describe why OR explain why blinding was not relevant to your study.*

Reporting for specific materials, systems and methods

We require information from authors about some types of materials, experimental systems and methods used in many studies. Here, indicate whether each material, system or method listed is relevant to your study. If you are not sure if a list item applies to your research, read the appropriate section before selecting a response.

Materials & experimental systems

n/a	Involved in the study	
☐	☒	Antibodies
☒	☐	Eukaryotic cell lines
☒	☐	Palaeontology and archaeology
☒	☐	Animals and other organisms
☐	☒	Human research participants
☒	☐	Clinical data
☒	☐	Dual use research of concern

Methods

n/a	Involved in the study	
☒	☐	ChIP-seq
☒	☐	Flow cytometry
☒	☐	MRI-based neuroimaging

Antibodies

Antibodies used	All the antibodies (primary antibodies for neuronal, vascular, and glial markers) and dyes used in the study are summarized in supplementary Table 1 of the paper.
Validation	The antibody validation were performed using the tranformation tissue protocol SHORT combined to advanced optical techniques, including confocal microscopy and light-sheet fluorescence microscopy.

Human research participants

Policy information about studies involving human research participants

Population characteristics	The brain tissue specimens were taken from 3 different human body donors with no known neuropsychiatric disorders
Recruitment	The brain tissue specimens were obtained from the body donation program of the Université de Tours and from the Massachusetts General Hospital (MGH)
Ethics oversight	The tissue donors gave their informed and written consent to the donation of their body for research purposes and they were obtained from the body donation program of the Université de Tours and from the Massachusetts General Hospital (MGH). Written consent was obtained from healthy participants prior to death, including the brain for any educational or research purposes. The authorization documents are kept with the MGH Autopsy Services in Boston, MA, and are available upon request. Collecting within the general frame of the approved IRB submission to the Partners Institutional Biosafety

Committee (PIBC., protocol 2003P001937), the tissue samples given by MGH Autopsy Services do not require a specific ethics approval documentation.

Note that full information on the approval of the study protocol must also be provided in the manuscript.
